# Poster Session I - A146 VILLAGE TO VISCERA: EVALUATING THE EFFECT OF SOCIAL SUPPORT ON INFLAMMATORY BOWEL DISEASE MANAGEMENT

**DOI:** 10.1093/jcag/gwaf042.146

**Published:** 2026-02-13

**Authors:** R Ahmed, T Iftikhar, K Skinner, F Noor, U Chinna, T Guzowski

**Affiliations:** Medicine, University of Saskatchewan, Saskatoon, SK, Canada; Medicine, University of Saskatchewan, Saskatoon, SK, Canada; Medicine, University of Saskatchewan, Saskatoon, SK, Canada; Medicine, University of Saskatchewan, Saskatoon, SK, Canada; Medicine, University of Saskatchewan, Saskatoon, SK, Canada; Medicine, University of Saskatchewan, Saskatoon, SK, Canada

## Abstract

**Background:**

Inflammatory bowel disease (IBD) imposes physical and emotional burdens that often extend beyond medical management. While social support is known to buffer stress in chronic illness, its measurable impact on disease activity and quality of life remains unclear.

**Aims:**

Examine the relationship between perceived social support and disease control in IBD patients.

**Methods:**

A convergent parallel mixed-method design. Quantitative:IBD patients (CD n = 44; UC n = 6) completed the *Multidimensional Scale of Perceived Social Support (MSPSS)* and the *Short Inflammatory Bowel Disease Questionnaire (SIBDQ)*. Disease activity was assessed by ultrasonographic indices- Simple Ultrasound Score for Crohn’s Disease and Milan Criteria for UC. Qualitative: Participants were categorized based on MSPSS scores and randomly selected for an interview exploring lived experiences of disease management, coping, and perceived support. Transcripts were analyzed for themes.

**Results:**

**Quantitative:**MSPSS scores mean 66.4 ± 14.0, with 76% categorized as high support, 20% moderate, and 4% low support. Subscale means were Family (22.4 ± 5.0), Friends 21.9 ± 4.9, and Significant Other 22.1 ± 5.3, each showing ceiling effects. The mean SIBDQ score was 12.5 ± 4.0 (IQR 10 –15.25). SIBDQ scores had a weak correlation with MSPSS total. The special person subcategory had a slight positive correlation r = 0.184 (p = 0.2, 95% Cl = -0.10 to 0.44). The SUS had a slight positive correlation with total MSPSS score (r = 0.129, p = 0.45, 95% Cl = −0.175 to 0.410). The Milan score had a slightly negative correlation (r = -0.240, p = 0.64).

**Qualitative:** Participants reported that fatigue, pain, and urgency disrupted daily life, but those with strong support described better adaptation. Emotional distress:Low-support patients expressed anxiety and hopelessness, whereas high-support individuals demonstrated acceptance and confidence. Isolation:Limited support led to concealment and social withdrawal; strong support fostered emotional safety. Resilience:High-support patients engaged in counseling and mindfulness; low-support participants lacked such tools and motivation.

**Conclusions:**

Perceived social support showed minimal direct correlation with disease activity. A limitation in the tests can explain this. Patients with robust support networks saw illness as manageable and maintained a sense of control despite persistent symptoms. In contrast, those lacking support experienced self-perpetuating isolation and emotional fatigue. Given the qualitative data, there likely is a correlation, but our available assessment tools may be limited in identifying it.

**Funding Agencies:**

None

